# Questionnaires and checklists for central auditory processing screening used in Brazil: a systematic review^☆^

**DOI:** 10.1016/j.bjorl.2018.05.003

**Published:** 2018-06-21

**Authors:** Francielli Loss Volpatto, Inaê Costa Rechia, Alexandre Hundertmarck Lessa, Cristina Loureiro Chaves Soldera, Maria Inês Dornelles da Costa Ferreira, Márcia Salgado Machado

**Affiliations:** a Universidade Federal de Ciências da Saúde de Porto Alegre (UFCSPA), Curso de Fonoaudiologia, Porto Alegre, RS, Brazil; b Universidade Federal de Santa Maria (UFSM), Curso de Fonoaudiologia, Santa Maria, RS, Brazil; c Universidade Federal do Rio Grande do Sul (UFRGS), Curso de Fonoaudiologia, Porto Alegre, RS, Brazil; d Faculdade Nossa Senhora de Fátima, Curso de Fonoaudiologia, Caixas do Sul, RS, Brazil

**Keywords:** Surveys and questionnaires, Auditory, Hearing tests, Auditory perception, Brazil, Inquéritos e questionários, Audição, Testes auditivos, Percepção auditiva, Brasil

## Abstract

**Introduction:**

The action of listening involves a complex interaction between the peripheral and central auditory systems. Central auditory processing disorder can be described as any problem in one or more auditory abilities. Literature reports that behavioral questionnaires and checklists can be applied to screen individuals at risk for central auditory processing disorder.

**Objective:**

To identify and analyze in the national literature questionnaires and checklists for the screening of central auditory processing available in Brazil for the Portuguese language.

**Methods:**

The research was carried out in electronic databases and “gray literature”. The search strategy was: “questionnaires OR surveys and questionnaires AND auditory OR hearing tests OR auditory perception AND Brazil”. The research was carried out between June and August of 2017. Study selection followed inclusion and exclusion criteria. The criteria adopted included Brazilian studies, without date and design restriction, that were carried out, translated, adapted and/or validated to Brazilian Portuguese or European Portuguese, as tools for central auditory processing screening. International studies that were not adapted to the Portuguese language were excluded, as well as the ones that were not available in full.

**Results:**

A total of 3664 publications were found and seven articles were selected for this systematic review, according to the established criteria.

**Conclusions:**

There is scarce national literature for central auditory processing screening and the only tool validated to Brazilian Portuguese, published as a monograph, is the auditory processing domains questionnaire. It is suggested that new studies with greater methodological stringency related to the processes of tool adaptation and validation be developed and published in the usual scientific databases, aiming at greater diffusion and clinical applicability.

## Resumo

**Introdução:**

O ato de ouvir envolve uma complexa interação entre os sistemas auditivo periférico e central. O transtorno do processamento auditivo central pode ser descrito como qualquer alteração em uma ou mais habilidades auditivas. A literatura refere que questionários e *checklists* comportamentais podem ser aplicados para triagem de indivíduos em risco para o transtorno do processamento auditivo central.

**Objetivo:**

Identificar na literatura nacional e analisar questionários e *checklists* para triagem do processamento auditivo central disponíveis no Brasil para a língua portuguesa.

**Método:**

Pesquisa realizada em bases de dados eletrônicas e “literatura cinza”. A estratégia de busca foi a seguinte: *questionnaires OR surveys and questionnaires AND auditory OR hearing tests OR auditory perception AND Brazil*. A pesquisa foi realizada de junho a agosto de 2017. A seleção dos estudos seguiu critérios de inclusão e exclusão. Os critérios adotados compreenderam estudos brasileiros, sem restrição de data e delineamento, que tenham sido elaborados, traduzidos, adaptados e/ou validados para o português brasileiro ou europeu, questionários e *checklists* como instrumentos para triagem do processamento auditivo central. Foram excluídos estudos internacionais não adaptados à língua portuguesa, bem como aqueles não disponíveis na íntegra.

**Resultados:**

Foram encontradas 3.664 publicações e sete trabalhos foram selecionados para esta revisão sistemática, conforme os critérios estabelecidos.

**Conclusões:**

Há escassa literatura nacional para triagem do processamento auditivo central e o único instrumento validado para o português brasileiro, publicado em formato de monografia, é o *Auditory Processing Domains Questionnaire*. Sugere-se que novos trabalhos com maior rigor metodológico nos processos de adaptação e validação dos instrumentos sejam desenvolvidos e publicados nas bases científicas usuais, com vistas à maior difusão e aplicabilidade clínica.

## Introduction

The action of listening involves a complex interaction between the peripheral and central auditory systems. In Brazil, since the 1990s, studies have been carried out focusing on Central Auditory Processing (CAP)^1^ – defined by the American Speech-Language Hearing Association (ASHA) as the efficiency and effectiveness by which the central nervous systems uses auditory information. To do so, a set of skills is required, which aims to meet, discriminate, recognize, store and understand sound information.^2^

Central Auditory Processing Disorder (CAPD) can be described as any alteration in one or more auditory abilities, namely: sound localization and lateralization, auditory discrimination, recognition of auditory patterns, temporal aspects of hearing (temporal resolution, masking, integration and ordering), figure-ground and auditory closure.^3^

Individuals with CAPD have difficulty hearing and/or understanding auditory information, even when their auditory thresholds are quantitatively normal. They may have several difficulties, such as understanding speech in noisy environments, following instructions, discriminating similar speech sounds, and often request the repetition of verbal information. Overall, morbidities can also be observed regarding spelling, reading, and writing.^4^

There is a significant association between CAPD and language disorders and school learning difficulties.^5^, ^6^, ^7^, ^8^, ^9^, ^10^ Therefore, the manifestations indicative of possible alterations are frequently observed in this period – corroborating the fact that the language and learning processes are complex and that there is an association between them and the integrity of the peripheral and central auditory pathways. Thus, it is understood that parents’ and teachers’ perceptions regarding the child's auditory behavior in different situations of daily life are extremely important to detect those children at potential risk for CAPD.

The American Academy of Audiology (AAA)^2^ and ASHA^11^ indicate that screening scales can be used to identify individuals at risk for CAPD, since family-based questionnaires and checklists are tools that can assist in providing information about individual's communication deficits and the impact on the daily life. Several questionnaires and behavioral checklists that assess hearing skills have been created and mentioned in the international literature, such as Children's Auditory Performance Scale (CHAPS),^12^ Screening Instrument for Targeting Educational Risk (SIFTER),^13^, ^14^ Test of Auditory Processing Skills – Revised (TAPS-R),^15^ Children's Home Inventory of Listening Difficulties (CHILD),^16^, ^17^ Fisher's Auditory Problems Checklist (FAPC),^18^ Auditory Processing Domains Questionnaire (APDQ),^19^ Listening Inventory for Education (LIFE),^20^ Listening Inventory for Education – Revised (LIFE-R),^21^ Scale of Auditory Behaviors (SAB),^22^ The Listening Inventory (TLI)^23^ and Evaluation of Children's Listening and Processing Skills (ECLIPS).^24^

The use of such tools has been discussed in the international literature for many years, and there have been divergences regarding their relevance and clinical applicability.^11^, ^25^, ^26^, ^27^ However, in the Brazilian literature, there is no published evidence on which tools are available for the Portuguese language and have been used in research,^1^, ^28^, ^29^, ^30^, ^31^, ^32^, ^33^, ^34^, ^35^, ^36^, ^37^, ^38^, ^39^ nor is there information regarding the degrees of sensitivity, specificity and the auditory abilities focused by each one.

Therefore, the objective of this systematic review was to identify and analyze in the national literature the questionnaires and checklists for CAP screening available in Brazil for the Portuguese language.

## Methods

The research question that guided the present study was: “Which questionnaires and checklists for CAP screening are available in Brazil for the Portuguese language?”.

Aiming to identify the studies in CAP screening through a questionnaire or checklist, scientific articles were searched in the following electronic databases: National Library of Medicine (PubMed), Scopus, Scientific Electronic Library Online (SciELO) and the Latin-American and Caribbean System on Health Sciences Information (LILACS).

As an alternative search source, we also used the “gray literature” – defined as any non-conventional print or electronic publication produced at all governmental, academic, and corporate levels.^40^ Thus, unpublished studies in scientific databases were also considered for the present study, such as monographs, theses and dissertations. The searches were performed in the Brazilian Digital Library of Theses and Dissertations (BDTD) and informally, including searches on the electronic search portals and the bibliographic references cited in the articles and in the “gray literature”, directly or indirectly related to the theme of this systematic review.

The descriptors used were selected from the exact descriptors and terms obtained through the Health Sciences Descriptors (DeCs), organized by the Virtual Health Library – Bireme – and the research strategy was as follows: Questionnaires OR Surveys and questionnaires AND Auditory OR Hearing tests OR Auditory Perception AND Brazil.

The studies were independently analyzed and selected by two examiners and, in case of divergence, consensus was sought. The following inclusion criteria were used: Brazilian studies, with no date or design restriction, that were created, translated, adapted and/or validated for Brazilian or European Portuguese, using questionnaires and checklists as tools for CAP screening. International studies not adapted to the Portuguese language were excluded, as well as those not available as full-text.

The collected data were analyzed and qualitatively compared regarding the following variables: general characteristics, target audience, age range of application, translation, adaptation and/or validation to Portuguese, focused listening skills, number of questions, correlation with the CAP tests, national studies published in full that used the tool and the degrees of sensitivity and specificity – in cases where validation occurred.

## Results

The search strategy occurred from June to August 2017. First, the search was carried out in the usual scientific electronic databases, resulting in 3378 published articles. Of these, 66 articles were excluded as they were duplicates in and among the databases themselves. Of the 3312 remaining studies for the initial analysis, 3311 studies were excluded because they did not address the object of this study and/or because they were international studies not adapted to the Portuguese language. Only one article,^1^ which adequately met the selection criteria for this systematic review, was included.

Using the same search strategy, a search was carried out in the “gray literature” through the Brazilian Digital Library of Theses and Dissertations (BDTD), resulting in 279 studies (169 dissertations and 110 theses), which were excluded as they did not assess the study theme.

**Figure 1:**
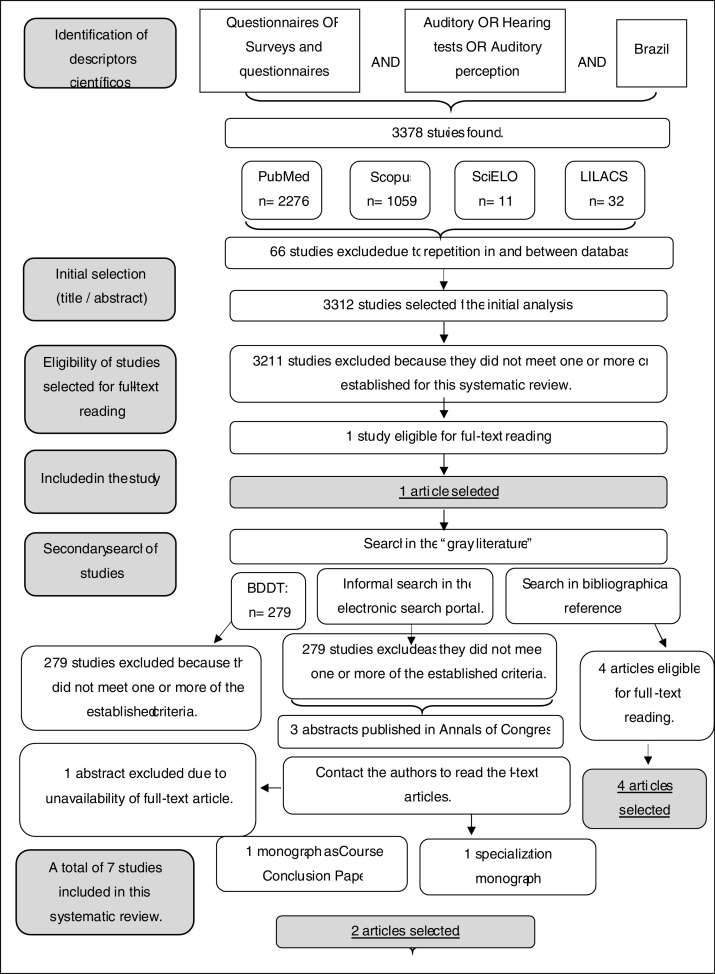
Diagram of study search and selection strategy.

Finally, informal searches were carried out in Google Scholar and the bibliographic references of the studies collected for this review. Therefore, four articles^35^, ^36^, ^37^, ^38^ and three abstracts^34^, ^41^, ^42^ were found, which had been published in Congress Annals that assessed the present theme. It was necessary to contact the authors to have access to the studies not available as full-texts. With the exception of one abstract,^34^ it was possible to obtain all studies in their original full-text versions. These search and selection strategies are shown diagrammatically in Fig. 1.

In total, seven articles were selected for this systematic review, as shown in Table 1. Of these, three questionnaires were informally created by the studies’ authors^36^, ^37^, ^38^; two were translated and adapted from the international literature^1^, ^43^ (SAB and CHAPS); one was translated only^35^; (QFISHER); and, finally, the other was translated, adapted and validated^44^ (APDQ). No original Brazilian studies that proposed to elaborate, adapt and validate tools to screen for CAP were identified.

**Table 1:** Studies selected for the analysis.

Title/author/year	Type of study	Objective of the study	Main findings
1. Otitis media and sound localization ability in preschool children. Lima-Gregio et al. (2010)^36^	Scientific article	To compare the performance of children in the SL test, with the parents’ answers to a questionnaire.	Except for the question that investigated inattention, there was no significant difference between the two tested groups – with the questionnaire and the SL test being insufficient tools to differentiate them. The authors believe this fact is justified by the socioeconomic factors of the assessed sample.
2. Identification of risk factors for the (central) auditory processing disorder in preschool children Luz and Costa-Ferreira (2011)^37^	Scientific article	To identify the risk factors for CAPD in preschool children attending the public school system.	The tool was effective in identifying statistically significant risk factors for CAPD in the studied population regarding the variables: greater number of siblings, greater request for repetition of instructions and comprehension difficulties in a competitive environment. Another important finding was the large number of children who never underwent any type of audiological examination, and some of them requested repetition of instructions frequently.
3. Perception of parents about the auditory attention skills of his kid with cleft lip and palate: retrospective study. Feniman et al. (2012)^38^	Scientific article	To verify the perception of the parents of children with CLP on their children's auditory attention.	The findings showed that most interviewed parents pointed out at least one of the attention-related behaviors included in the questionnaire, suggesting that the presence of CLP may be related to difficulties in auditory attention.
4. Scale of Auditory Behaviors and auditory behavior tests for auditory processing assessment in Portuguese children Nunes et al. (2013)^1^, ^22^	Scientific article	To investigate the hearing abilities of Portuguese children and to verify if there is correlation between them and the Scale of Auditory Behaviors (SAB) score.	A significant correlation was observed between the questionnaire score and that of the behavioral tests, the highest of which was observed in the tests related to temporal processing. The higher the SAB score, the better the responses obtained at the behavioral evaluation of the CAP. The study also confirmed that most children with a score <46 points in the SAB had an alteration in one or more of the CAP tests.
5. The auditory processing domains questionnaire (APDQ): Portuguese version. Yokoyama, et al. (2015)^19^, ^33^, ^44^	Monograph	To adapt the APDQ into Portuguese and apply the Portuguese version of the questionnaire to a group of school-aged children without CAP alterations.	A Portuguese version of the APDQ questionnaire was obtained through the translation and validation processes. The total score of the original tool is 208 points and the Brazilian version has a total of 199.2 – very close to the maximum score of the original version. The tool was validated with 100% of sensitivity and specificity.
6. Questionnaire children's auditory performance scale: translation and adaptation into Brazilian Portuguese Donadon et al. (2015)^12^, ^43^	Monograph	To translate and culturally adapt the CHAPS questionnaire into Brazilian Portuguese and apply it to verify its effectiveness.	The tool went through all stages of the cultural adaptation process, obtaining substantial support for its content validity according to semantic-idiomatic and cultural equivalence criteria. The members of the committee agreed on 84% with the cultural adaptation of the questionnaire. There was a correlation between the questions of the “Auditory Memory” module and the performance in the DD test – binaural integration step.
7. Use of questionnaires in the monitoring of auditory training results. Cibian and Pereira (2015)^18^, ^35^	Scientific article	Monitor auditory behavior through Fisher's Auditory Problems Checklist in 19 individuals diagnosed with CAPD who underwent auditory training.	The sample, who showed alterations in selective attention and/or temporal processing skills, were submitted to auditory training and demonstrated, midway and at the end of the training, an improvement in the score of the questionnaire answered by the parents.

SL, sound location; ME, middle ear; CAPD, central auditory processing disorder; CLP, cleft lip and palate; CAP, central auditory processing; DD, dichotic digit test.

Table 2 shows the population groups that have been previously studied and the respective questionnaires that have been used.

**Table 2:** Description of population groups assessed by questionnaire.

PG	Mean age range	Questionnaire Lima-Gregio et al.^36^	Audiological and cognitive aspects in pre-schoolers^37^	Auditory/Attention Questionnaire^38^	SAB^1^, ^22^	APDQ^19^, ^33^, ^44^	CHAPS^12^, ^43^	QFISHER^18^, ^35^
Individuals with morbidities and/or CAPD	6–17 years	X	X		X	X	X	X
Individuals with CLP	6–11 years			X			X	
Individuals with OSAS	6–12 years				X			

PG, population groups; CAPD, central auditory processing disorder; CLP, cleft lip and palate; OSAS, obstructive sleep apnea syndrome.

Table 3 demonstrates the auditory skills assessed by each tool.

**Table 3:** Auditory skills contemplated by each questionnaire.

Instrumentos	Auditory skills					
	Figure-ground and auditory closure	Auditory attention	Binaural interaction	Temporal resolution	Temporal ordering	Binaural integration and separation
Questionnaire by Lima-Gregio et al.^36^	X	X	X			X
Audiological and Cognitive Aspects in Pre-Schoolers^37^	X	X		X		X
Auditory/Attention Questionnaire^38^	X	X		X	X	
SAB^1^, ^22^	X	X		X	X	X
APDQ^19^, ^33^, ^44^	X	X	X	X	X	X
CHAPS^12^, ^43^	X	X	X	X	X	X
QFISHER^18^, ^35^	X	X		X	X	X

Table 4 compares and synthesizes the tools regarding the analyzed variables.

**Table 4:** Comparison of questionnaires and checklists for screening of central auditory processing.

Version available in Brazil	Current situation	Tool characteristics	Target audience and application range	Type and number of questions	Correlations with CAP tests	Other studies that used the tool
Lima-Gregio et al. (2010) Questionnaire^36^	National; non-validated.	Covers basic audiological complaints such as hearing, otitis and ME alterations, as well as risk factors and classic signs and symptoms of CAPD; Does not generate a score.	**Target audience:** Parents; **Range of application:** Preschoolers.	14 mixed questions (open and closed)	No correlation was found with the SL test.^36^	Not found
Audiological and Cognitive Aspects in Pre-Schoolers (Luz and Costa-Ferreira, 2011)^37^	National; non-validated.	Covers several aspects of child development: gestation, birth, development, family history, routine, language, school learning and auditory behaviors suggestive of CAPD. Does not generate a score.	**Target audience:** Parents; **Range of application:** Preschoolers and 1st-Grade students.	39 mixed questions (open and closed)	No such correlations were carried out.	Not found
Auditory/Attention Questionnaire (Feniman et al., 2012)^38^	National; non-validated.	It is divided into three parts: Part I (Child Identification), Part II (Hearing Health) and Part III (Child's attention); It covers the following aspects of hearing health: history of hearing loss and ME infections. Contains a checklist with aspects related to hearing health. Does not generate a score.	**Target audience:** Parents; **Range of application:** 6–11 years of age.	Part I: 8 open questions; Part II: 2 mixed questions (open and closed); Part III: 32 closed questions. Total: 42 questions	No such correlations were carried out.	Not found
Scale of Auditory Behaviors – SAB (Nunes et al., 2013)^1^	Translated and adapted to European Portuguese.	Likert scale style; It covers items of auditory behavior most frequently related to CAP; Generates score: The sum of the items generates a final score and, according to the performance, it indicates: typical auditory behavior for the age group; need to evaluate CAP; or probable alteration in CAP.	**Target audience:** Parents and/or teachers **Range of application:** 10–13 years of age	12 closed questions.	There was correlation with all eight tests applied; statistical significance for the following testes: MSV, MSNV; FR in LE; DD in RE; TDDH in the RE and LE; DP; GIN in the RE and LE. The highest correlation occurred in the PD test.^1^	Kemp AAT, Cardoso ACV (2016)^29^; Leite Filho CA, et al. (2017)^28^
Children's Auditory Performance Scale – CHAPS (Donadon et al., 2015)^43^	Translated and adapted to Brazilian Portuguese.	Likert scale style; Divided into six Auditory Task modules: In Noise, In Silence, In ideal Condition, Multiple Information, Auditory Memory/Sequencing and Extended Auditory Attention. Generates score: Gross and average, being possible to analyze them by module or by the total sum. According to the performance, it indicates normal individuals or those at risk for the CAPD. Analysis of results and reference values are not described in the translation and adaptation study.	**Target audience:** Parents and/or teachers **Range of application:** 7–14 years of age.	36 closed questions.	There was correlation with the DD Test (binaural integration step).^43^	Manoel and Freitas (2006)^31^ Barufi et al. (2004)^39^ Manoel et al. (2010)^30^
Fisher's Auditory Problems Checklist for Auditory Processing Evaluation – QFISHER (Cibian e Pereira, 2015)^35^	Informally translated into Brazilian Portuguese.	Likert scale style; The questions cover hearing, attention, memory, language and school performance aspects. Generates a score: The sum of the items generates a score by category (Hearing, Attention, Memory, Language and School Performance) and a total score. The individual is considered to be at risk for the CAPD if 7 or more items are scored.	**Target audience:** Parents and/or teachers **Range of application:** 12–15 years of age.	24 closed questions.	There was improvement of the scores after auditory training of dichotic approach with the following tests: DD, NVD, PSI, SSI, DCVT and LSPMC^35^	Geribola and Lewis (2008)^32^
Auditory Processing Domains Questionnaire – APDQ (Yokoyama et al., 2015)^44^	Translated and validated to Brazilian Portuguese with 100% sensitivity and specificity.	Likert scale style; It has a directed anamnesis; It covers the everyday life auditory skills of a student: decoding, prosody, auditory separation and binaural integration, taking into consideration the quiet and noisy environment. It also includes attention, language and school aspects. Generates score: Analysis performed according to three subscales: “Auditory Processing”, “Attention Control” and “Linguistic and Cognitive Skills”; Analysis of the results and parameters of normality were not described in the translation, adaptation and validation study.	**Target audience:** Parents and/or teachers **Range of application:** 7–17 years.	52 closed questions.	The following CAP tests were carried out: LS, MSV, MSNV, DP, RGDT, SSI, IPRF, FR, DD, DNV (free and targeted attention). There was correlation with the SL test.^33^	Martins KVC et al. (2015)^33^

CAP, central auditory processing; ME, middle ear; CAPD, central auditory processing disorder; SL, sound location; VSM, verbal sequential memory; NVSM, non-verbal sequential memory; SIN, speech-in-noise; LE, left ear; DD, dichotic digit test; RE, right ear; TDDH, harmonic pattern dichotic digits test; DP, duration pattern; GIN, gap-in-noise; RGDT, random gap detection test; SSI, synthetic sentence identification; PISR, percentage index of speech recognition; NVD, non-verbal dichotic test; PSI, pediatric speech intelligibility; DCVT, dichotic consonant–vowel test; LSPMC, list of sentences in Portuguese with contralateral message.

## Discussion

Questionnaires are important tools in clinical practice and in several fields of knowledge, and currently there are few tools available in Brazilian Portuguese for audiology.^43^ Regarding the tools for CAP screening, the interest in the creation, translation, adaptation and/or validation of these tools in Brazil is quite recent, compared to the international scientific productions, all emerging in the last decade.

Regarding the translation, adaptation and/or validation studies, it is important to highlight the difficulty of locating them in the scientific databases. With the exception of the Scale of Auditory Behaviors (SAB),^1^ published in article format, the remaining articles were found only by searching the “gray literature”. It is believed that this fact influences the small number of studies found at the national level that have used questionnaires and checklists as CAP function screening methods^28^, ^29^, ^30^, ^31^, ^32^, ^33^, ^39^ and, consequently, the possible lack of knowledge by professionals regarding the use of such tools in clinical practice.

The population assessed by the tools found in the present systematic review comprises children and adolescents, with a predominance of preschoolers and school-aged children. This was also observed in seven other studies^28^, ^39^ that used these diagnostic tools in their methodologies for CAP assessment, and it was observed that CHAPS and SAB were the most often used questionnaires in the national literature.^28^, ^39^

The difficulty in identifying and/or the absence of screening tools in questionnaire or checklist format aimed at adult and elderly individuals is emphasized. This is believed to be due to the fact that the signs and symptoms of CAPD manifest mainly during the school period, making the study focus aim at the pediatric population for early detection and intervention.^5^, ^6^, ^7^, ^8^, ^9^, ^10^ A positive association between CAPD and reading/writing learning difficulties/disorders has been well established in the literature. Therefore, it is emphasized that losses in such processes can jeopardize an adequate overall child development.

Regarding the number of questions, this variable was very heterogeneous. For purposes of classification, in this systematic review, a “short tool” was defined as that having 15 items and a “long tool” as having more than 15 items. With the exception of two questionnaires,^1^, ^36^ the rest of the assessed tools are long, containing an average of 38 questions. In clinical practice, the application time of the tool is also a variable to be considered and, in the identified studies, this factor was not explained.

Not all studies sought to associate performance in the questionnaire and CAP tests. Therefore, the auditory abilities focused on each one were studied also considering the subjective analysis of the items of each tool, according to Table 3. It can be observed that the tools that cover all CAP auditory abilities are the APDQ and CHAPS. However, more studies are needed for objective verification.

Questionnaires such as the one by Lima-Gregio et al.,^36^ “Audiological and Cognitive Aspects in Preschoolers”^37^ and the “Auditory/Attention Questionnaire”,^38^ were created as methodological tools of their respective studies.

The questionnaire created by Lima-Gregio et al.^36^ aimed to compare the performance of children with and without a history of recurrent otitis media, in the Sound Localization test (SL), with parents’ answers to a questionnaire. With the exception of the question that investigated inattention, there was no significant difference between the two tested groups – the questionnaire and the SL Test were insufficient tools to differentiate the tested groups. The authors believe this fact is justified by socioeconomic factors of the assessed sample.^36^

On the other hand, the questionnaire called “Audiological and Cognitive Aspects in Preschoolers”^37^ aimed to identify the risk factors for CAPD in preschool children attending the public school system. The tool was effective in identifying statistically significant risk factors for CAPD in the assessed population regarding the variables: higher number of siblings, more requests to repeat instructions and difficulty in understanding in a competitive environment. Another important finding was the high number of children who had never been submitted to any type of audiological examination, with some of them often requesting repetition of instructions.^37^

The tool “Auditory/Attention Questionnaire”^38^ was used to assess children with cleft lip and palate (CLP) through the parents’ perception. The findings showed that most of the interviewed parents pointed out at least one of the behaviors related to attention contained in the questionnaire, suggesting that the presence of CLP may be related to difficulties in hearing care.^38^ Although that is the main focus of the tool, it can be observed that several questions include more skills, which suggests it can be used for other skills in addition the auditory attention and in other populations, as well as the fact that it can identify or screen individuals with Attention-Deficit and Hyperactivity Disorder (ADHD).

The SAB tool, originally proposed by Schow, Seikel, Brockett and Whitaker in 2007,^22^ was adapted and translated into European Portuguese.^1^ The important correlation between the tool and all the auditory abilities tested in the study is highlighted, but mainly with the ability of temporal ordering. In Brazil, some studies have been used to investigate auditory behavior and temporal resolution of children with obstructive sleep apnea syndrome (OSAS)^28^ and CAP function in students in the first years of schooling.^29^ It should be noted that there is a published version of SAB available for Brazilian Portuguese,^45^ which, however, does not show methodological data for the translation, adaptation and validation steps of the tool for use in this population.

The CHAPS tool^12^ has been translated and adapted into Brazilian Portuguese.^43^ During the process of translation and adaptation to Brazilian Portuguese, there was a correlation between the Auditory Memory module questions and the performance in the Dichotic Digits Test (DD) – binaural integration step. Brazilian studies used CHAPS prior to its translation and formal adaptation, aiming to assess the auditory behavior of children submitted to surgery due to CLP through the assessment of teachers^30^, ^31^ and the parents’ own perception.^39^

CHAPS is the tool most often used in the assessment of children with CLP.

The FAPC tool (1976)^18^ does not have an adapted version, but has been informally translated in studies^32^, ^35^ to be used as a screening tool for CAPD in children. The tool is a scale and consists of 24 questions that address the behavioral difficulties observed in the individual's daily life. If the scoring of the items is equal to or greater than seven, the individual is considered to be at risk for CAPD.

The use of the FAPC, called by the study authors^35^ as “QFISHER”, is highlighted as an effective tool for auditory training monitoring in children with CAPD. The study demonstrated an association between the improvement in the scores with the following tests: DD, Non-verbal Dichotic (NVD), Synthetic Sentence Identification with Competitive Message (PSI/SSI), Dichotic Consonant-Vowel (DCV) and List of Sentences in Portuguese with contralateral message (LSPMC).

It is noteworthy that the APDQ questionnaire^19^ was translated and validated into Brazilian Portuguese with 100% sensitivity and specificity, being therefore the most robust tool for use in clinical practice and research. One study^44^ used the questionnaire after it was translated, back-translated and culturally adapted in a group of school-age children without CAPD. The total score of the original questionnaire was 208 points and the translated version averaged 199.2 points – close to that of the original score. Another study^33^ used the translated and validated version in school-age individuals with CAPD, obtaining an average of 92.6 points. The authors state that there is a statistically significant difference between the CAPD and the non-CAPD groups assessed in the translation study, suggesting this questionnaire can be a potential tool to identify individuals at risk. Nevertheless, there was a positive correlation between the total score of the questionnaire and the SL Test.

The information obtained through well-structured questionnaires about the child's auditory behavior by third parties, such as parents, guardians and/or teachers, can be very useful for the early identification of changes in CAP function, which will lead to other evaluation, diagnostic and therapeutic processes that will ensue, according to each child's needs. However, more national studies should be developed to demonstrate their effectiveness, mainly by associating the screening tools to the CAP behavioral evaluation data.

This systematic review shows the need to develop screening tools for CAP function for the adult and elderly populations, as they are currently restricted to the pediatric population. Regarding the study translation, adaptation and/or validation processes, it is essential to clarify the analysis of the results and parameters of normality of each tool for the Brazilian population, since these limitations were found in most studies found in the “gray literature”.

New studies with greater methodological stringency should be carried out and published for the purpose of demonstrating reliability, supporting evidence-based practice and disseminating the use another auxiliary tool in the diagnosis of CAPD, which will allow the use of such tools in clinical practice.

## Conclusion

There is a scarcity of national literature on CAP screening and the only tool validated to Brazilian Portuguese, published as a monograph, is the Auditory Processing Domains Questionnaire (APDQ). It is suggested that new studies with greater methodological stringency related to the processes of tool adaptation and validation be developed and published in the usual scientific databases, aiming at greater diffusion and clinical applicability.

## Conflicts of interest

The authors declare no conflicts of interest.
